# The Releasate of Avascular Cartilage Demonstrates Inherent Pro-Angiogenic Properties *In Vitro* and *In Vivo*

**DOI:** 10.1177/19476035211047628

**Published:** 2021-09-30

**Authors:** Yannick Nossin, Eric Farrell, Wendy J.L.M. Koevoet, Frank Datema, Rodrigo A. Somoza, Arnold I. Caplan, Gerjo J.V.M. van Osch

**Affiliations:** 1 Department of Otorhinolaryngology, Erasmus MC, University Medical Center Rotterdam, Rotterdam, the Netherlands; 2 Department of Oral and Maxillofacial Surgery, Erasmus MC, University Medical Center Rotterdam, Rotterdam, the Netherlands; 3 Department of Biology, Skeletal Research Center, Case Western Reserve University, Cleveland, OH, USA; 4 CWRU Center for Multimodal Evaluation of Engineered-Cartilage, Cleveland, OH, USA; 5 Department of Orthopaedics, Erasmus MC, University Medical Center Rotterdam, Rotterdam, the Netherlands; 6 Department of Biomedical Engineering, Faculty of Mechanical, Maritime, and Materials Engineering, Delft University of Technology, Delft, the Netherlands

**Keywords:** cartilage, chondrocyte, angiogenesis, functional assays

## Abstract

**Objective:**

Cartilage is avascular and numerous studies have identified the presence of single anti- and pro-angiogenic factors in cartilage. To better understand the maintenance hyaline cartilage, we assessed the angiogenic potential of complete cartilage releasate with functional assays *in vitro* and *in vivo*.

**Design:**

We evaluated the gene expression profile of angiogenesis-related factors in healthy adult human articular cartilage with a transcriptome-wide analysis generated by next-generation RNAseq. The effect on angiogenesis of the releasate of cartilage tissue was assessed with a chick chorioallantoic membrane (CAM) assay as well as human umbilical vein endothelial cell (HUVEC) migration and proliferation assays using conditioned media generated from tissue-engineered cartilage derived from human articular and nasal septum chondrocytes as well as explants from bovine articular cartilage and human nasal septum. Experiments were done with triplicate samples of cartilage from 3 different donors.

**Results:**

RNAseq data of 3 healthy human articular cartilage donors revealed that the majority of known angiogenesis-related factors expressed in healthy adult articular cartilage are pro-angiogenic. The releasate from generated cartilage as well as from tissue explants, demonstrated at least a 3.1-fold increase in HUVEC proliferation and migration indicating a pro-angiogenic effect of cartilage. Finally, the CAM assay demonstrated that cartilage explants can indeed attract vessels; however, their ingrowth was not observed.

**Conclusion:**

Using multiple approaches, we show that cartilage releasate has an inherent pro-angiogenic capacity. It remains vessel free due to anti-invasive properties associated with the tissue itself.

## Introduction

Cartilage is an avascular tissue and the absence of blood vessels is considered a key feature in the homeostasis of permanent cartilage.^1^ Healthy adult cartilage is often assumed to be anti-angiogenic in nature. Indeed, it was shown that cartilage explants or tissue-engineered cartilage constructs from adult chondrocytes are not invaded by blood vessels and retains its stable cartilage phenotype when implanted subcutaneously in mice.^2,3^ Eisenstein and colleagues have demonstrated that cartilage inhibits vessel invasion^4^ due to factors that can be extracted from the tissue with guanidine hydrochloride.^5^ Since components in this extracted fraction could be a useful tool against cancer (anti-invasion factors),^6,7^ this further prompted search for specific proteins in cartilage that conferred the tissue with this anti-angiogenic capacity and led to the identification of proteins, such as chondromodulin and endostatin, that prevented vessel formation and invasion.^6-13^

However, cartilage does become invaded with blood vessels and can undergo endochondral ossification in early stages of limb development or when its integrity is disrupted as seen in osteoarthritis (OA).^14,15^ Most works studying the progression OA have found prevalent pro-angiogenic factors, most notably one of the best-known factors driving angiogenesis, vascular endothelial growth factor (VEGFa).^16^ Interestingly, VEGFa is not only expressed in OA cartilage but also in mature healthy cartilage and chondrocyte-derived tissue-engineered constructs^17-19^ that will not be invaded by blood vessels. A pro-angiogenic potential of cartilage has been observed in experiments with chondrocytes forming networks *in vitro* in Matrigel^20,21^ and with conditioned medium of cultured chondrocytes on human umbilical vein endothelial cell (HUVEC) proliferation, migration, and tube formation *in vitro*.

These apparent discrepancies reported in the literature about pro- and anti-angiogenic properties of articular cartilage led us to question the true angiogenic potential of cartilage. We set out to analyze the angiogenesis regulating gene expression of cartilage, and performed functional assays *in vitro* and *in vivo* to evaluate the angiogenic effect of factors released from tissue engineered cartilage as well as cartilage explants.

## Methods

### Generation of Conditioned Medium from Cartilage Explant and Tissue-Engineered Constructs

Articular cartilage was obtained from 6 patients (3 males, 3 females, age 63-86 years) undergoing total knee replacement surgery with implicit consent of the use of leftover material after surgery (after approval by local ethics committee; MEC-2004-322). Cartilage was taken from macroscopically unaffected areas. Nasal cartilage was obtained from 7 patients (3 males, 4 females, age 17-65 years) undergoing septal corrections with implicit consent. Perichondrium was removed from nasal cartilage with a scalpel. Four nasal septal cartilage donors were utilized for chondrocyte isolation (2 males, age 58-65 years; 2 females, age 17-21 years). To isolate chondrocytes, harvested cartilage was treated with 2 mg/mL protease B in physiological saline solution (Sigma-Aldrich, St. Louis, MO, USA) for 90 minutes and subsequently digested overnight in basal medium (Dulbecco’s modified Eagle medium [DMEM], 4.5 g/L glucose with 10% fetal calf serum [FCS], 50 µg/mL gentamicin, and 1.5 µg/mL fungizone [all Invitrogen, Carlsbad, CA, USA]) supplemented with 0.12 U collagenase B (Roche Diagnostics, Almere, the Netherlands). The resulting primary chondrocytes were seeded at a density of 7,500 cell/cm^2^ in T175 culture flasks for expansion with the above-mentioned basal medium. For generation of stable cartilage pellets, articular chondrocytes at passage 1 and nasal chondrocytes at passage 3 were utilized.

Pellet cultures of chondrocytes were formed by seeding 2.0 × 10^5^ cells in 0.5 mL in a 15-mL conical polypropylene tube and centrifuging for 8 minutes at 300 × *g*. Pellets from both cell sources were cultured in normoxic conditions for 21 days in chondrogenic medium (high-glucose DMEM with 50 μg/mL gentamicin [Invitrogen], 1.5 μg/mL fungizone ([nvitrogen], 1 mM sodium pyruvate [Invitrogen], 40 μg/mL proline [Sigma, Kawasaki, Kanagawa Prefecture, Japan], 1:100 v/v insulin-transferrin-selenium [ITS; BD Biosciences, San Jose, CA, USA], 10 ng/mL transforming growth factor β1 [TGFβ1; R&D Systems], 10 mM ascorbic acid-2-phosphate [Sigma], and 100 nM dexamethasone [Sigma]). The medium was renewed twice a week. At day 21, medium was renewed and 24 hours later the pellets were washed with phosphate buffered saline (PBS) and incubated with basal medium consisting of phenol-red free DMEM (Gibco, Waltham, MA, USA) with 0.1% w/v bovine serum albumin (BSA; Sigma) and 10 mM ascorbic acid-2-phosphate (Sigma) for 24 hours to produce conditioned medium (CM) for downstream experiments. This CM was collected, cell debris removed by centrifugation at 300 × *g* for 8 minutes and stored at −80 °C. Pellets were digested in 350 μL RNABee (Tel-Test, Inc., Pearland, TX, USA) and stored at -80°C for subsequent gene expression analysis. Additional pellets were fixed in 4% formalin at room temperature overnight and then processed for histological analysis.

To generate CM from tissue explants we used nasal septal cartilage (3 donors) and 3 bovine fetlock joints as source for hyaline cartilage. After determining their weight and washing with basal medium, the explants were placed per 100 mg of tissue in 1 ml of basal medium. After 24 hours the CM was collected, cell debris removed by centrifugation at 300 × *g* for 8 minutes and stored at −80 °C.

### Identification of Angiogenesis Regulating Genes in RNAseq Dataset

To identify known angiogenesis regulating factors expressed by human adult articular cartilage using a dataset generated previously^22^ (GSE128554). More details can be found in the Supplemental Material. The data were compared with Gene Ontology and Uniprot data regarding: angiogenesis (GO:0001525) 604 genes, negative regulation of angiogenesis (GO:0016525) 91 genes, as well as positive regulation of angiogenesis (GO:0045766) 218 genes.

### Gene Expression Analysis

To isolate RNA, the pellets in RNABee were homogenized with an Eppendorf Micro-pestle (Eppendorf, Hamburg, Germany). Total RNA isolation was performed utilizing the RNeasy Column system (Quiagen, Hilden, Germany). 0.5 μg RNA was used for cDNA synthesis using the RevertAid First Strand cDNA kit (Thermo Fisher, Waltham, MA, USA). Gene expression was analyzed by real-time reverse transcription quantitative polymerase chain reaction (RT-qPCR) on a StepOnePlus System using SYBR Green (Applied Biosystems, Foster City, CA, USA) or Taqman (Thermo Fisher) assays. Primer and probe sequences in the supplementary materials. The best housekeeper index (BKI) was calculated from *GAPDH*, *RSP27*, and *HPRT*.

### Histology

Fixed pellets were paraffin embedded and sectioned. Deposition of glycosaminoglycan (GAG) was determined by thionine staining.^23^

For immunohistochemical stainings, sections were pretreated with 0.1% w/v pronase and 1% w/v hyaluronidase and blocked using normal goat serum (Southern Biotech, Birmingham, AL, USA). Sections were incubated with either mouse monoclonal antibody against collagen type II 0.4 μg/mL (Developmental Studies Hybridoma Bank, Cat. #II-II6B3) for 60 minutes or collagen type X 5 μg/mL (ThermoFisher, Clone X53, Cat. #14-9771-82) in PBS 1% BSA overnight. After incubation with a biotinylated goat anti-mouse antibody and ALP-conjugated streptavidin, staining was revealed by incubation with a New Fuchsin substrate (Chroma, Kongen, Germany). As corresponding isotype controls 0.4 μg/mL and 5 μg/mL of an isotype immunoglobulin G1 monoclonal antibody were used.

### Angiogenesis Assays

Commercially derived pooled HUVEC (Lonza, Basel, Switzerland) were seeded at a density of 5 × 10^3^ cells/cm^2^ in culture flasks and cultured in endothelial growth medium (EGM-2 Promocell, Heidelberg, Germany). The medium was renewed every 2 to 3 days. When they neared confluency, cells were detached with 0.05 % trypsin-EDTA (Gibco) and used for angiogenesis assays. For angiogenesis assays, HUVECs between passages 8 and 10 were used.

### Endothelial Cell Migration Assay

Migration assays were performed by seeding HUVEC (5 × 10^4^ cells/well) in 24-well Transwell inserts (8 µm pore size, Corning Life Sciences, Corning, NY, USA) in serum-free medium containing 0.05% BSA (Merk, Kenilworth, NJ, USA). The different CMs were placed in the lower compartment of different wells and diluted 1:1 with endothelial basal medium (EBM-2, Promocell). EBM and unconditioned medium (UCM) were used as negative controls and EGM-2 was used as positive control. After 10 hours of incubation at 37 °C and 5% CO_2_, the cells on the membrane were fixed with 4% formaldehyde/PBS and the nonmigrated cells from the upper surface of the membrane were removed with a cotton swab. We confirmed that no cell proliferation took place during the 10-hour incubation, based on cell count. The migrated cells were then stained with DAPI (4′,6-diamidino-2-phenylindole) and then quantified by fluorescence microscopy and image analysis through ImageJ utilizing the included particle analysis macro. Five nonoverlapping pictures were taken and the average cell count per well for three independent experiments was calculated.^24^

### Endothelial Proliferation

To measure proliferation, 2.5 × 10^3^ HUVEC/cm² were seeded in 48-well plates. After 24 hours, the medium was replaced with EBM to synchronize the cells. After 8 hours, the cells were stimulated with CM, EGM-2 as positive control, or EBM-2 as negative control for 24 hours. With the stimulus, 10 µM dEdU (d-5-ethynyl-2′-deoxyuridine; BaseClick, Neuried, Germany) was added to stain DNA of replicating cells. After 24 hours, the cells were washed with PBS and fixed with 4% formalin for 5 minutes. The EdU-label was revealed utilizing the manufacturers protocol. The cells were counterstained with DAPI and imaged using fluorescence microscopy. Utilizing the particle analysis macro in ImageJ, we determined the average amount of positively stained cells per image for DAPI and EdU and the percentage of cells positive for EdU was calculated.

### Chick Chorioallantoic Membrane Assay

Fertilized chicken eggs laid the day before (purchased from Drost Loosdrecht B.V., Netherlands) were incubated sideways at 37 °C and 65% humidity for 3 days before rupturing the air-sack and opening a small hole on the top to deflate the air-sack. At day 7 of incubation, part of the shell was removed to start the assay. CM was concentrated 20-fold using Amicon Ultra-2 mL Centrifugal Filters (Merck, Kenilworth, NJ, USA). One sterile filter disk (5 mm diameter) soaked with 5 µL concentrated CM was placed on the chorioallantoic membrane (CAM) of the egg. Concentrated basal medium was used as negative and 100 ng FGF2 was used as positive control.^25^ After 3 days of incubation, the CAM was fixed with 4% formalin and removed from the egg. Induction of vessel formation was assessed by 3 independent observers ranking blinded 45 pictures for the amount of attracted vessels and their directionality toward the stimulus.

Eight bovine cartilage explants (5 mm diameter) were placed on filter rings on the CAM of 8 separate eggs. After 7 days of incubation the tissue and surrounding CAM were fixed with 4% formalin for 24 hours, imaged, and subsequently processed for histology.

### Data Analysis and Statistics

Before each statistical test the data were evaluated for normal distribution via descriptive statistics as well as visual inspection and subsequently the appropriate tests were chosen. The *in vitro* angiogenesis assays were statistically evaluated with a linear mixed model (experimental conditions as fixed factor, repeats as random factor) with Bonferroni *post hoc* correction. For articular chondrocytes constructs 2 batches of CM created by pooling 3 donors were tested in triplicate in independent experiments. For the cartilage explant derived CM as well as the nasal chondrocyte constructs CM of each donor was tested separated in triplicate. For the CAM assay, the interobserver correlation was tested through a Spearman correlation test. The conditions were compared using the average rank of 3 observers with a Mann-Whitney *U* test. Data are described as mean and standard deviation. The positive controls, included to be able to exclude technical failures, were calculated separately from statistical analyses. Statistical analysis was performed using SPSS 11 for Windows (IBM, Armonk, NY, USA).

## Results

### Gene Expression Analysis Revealed Plethora of Pro-Angiogenic Genes Expressed by Healthy Adult Cartilage

**Figure 1.**
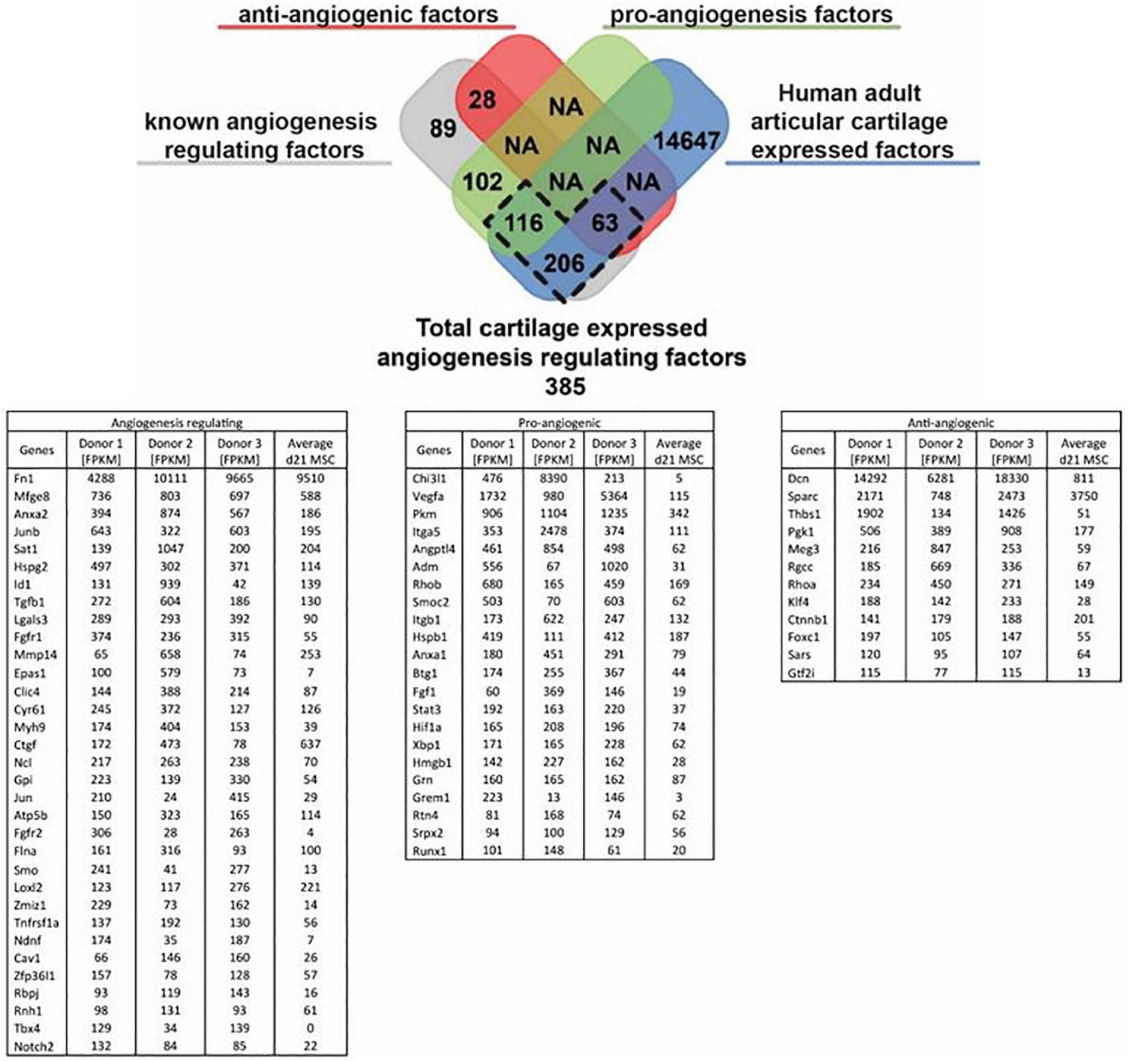
Expression of angiogenesis regulating genes in human adult articular cartilage. Table depicts the angiogenesis regulating fraction of the top 1000 highest expressed human adult articular cartilage (HAAC) genes as well the average of chondrogenically differentiated mesenchymal stem cells (MSCs) of 3 donors as a known pro-angiogenic condition for comparison. FPKM = fragments per kilobase of transcript per million mapped reads.

In healthy adult human articular cartilage 385 of the total 15032 expressed genes were associated to regulation of angiogenesis or vasculogenesis. Of these 385 genes 63 genes were associated with the negative regulation of angiogenesis and 116 were known pro-angiogenic genes of which most are more highly expressed than d21 chondrogenic differentiated mesenchymal stem cells (MSCs)^26^ (**Fig. 1**).

### Releasate of Tissue-Engineered Cartilage Constructs Promoted Endothelial Cell Proliferation and Migration *In Vitro* and Angiogenesis *In Vivo*

**Figure 2.**
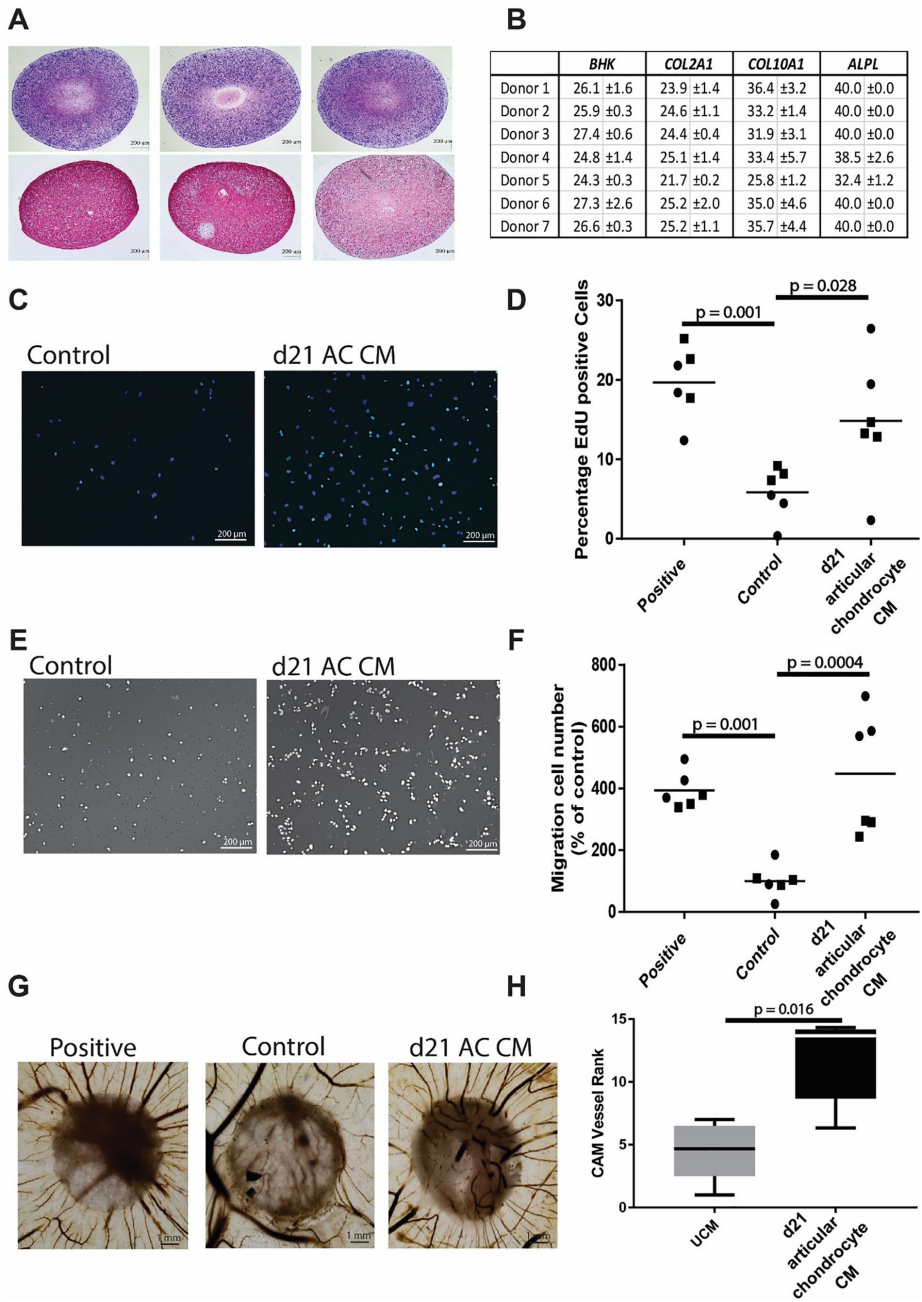
Pro-angiogenic effect of conditioned medium from cartilaginous tissue generated from culture expanded human articular chondrocyte. (**A**) Histological and immunohistological staining of day 21 chondrogenically redifferentiated chondrocyte pellets of 3 donors for thionine (first row) and collagen type II (second row). (**B**) Quantitative polymerase chain reaction (qPCR) gene expression analysis of chondrogenic and hypertrophy marker genes showing the mean and SD of 3 donors. Cutoff value for expression Ct = 35, BHK = best housekeeper index calculated from GAPDH, RSP27, and HPRT1. (**C**) Endothelial proliferation assay, showing in cyan positive EdU staining, counterstained with DAPI (blue). (**D**) Quantification of proliferating HUVECs after 24 hours. (**E**) Endothelial migration assay. (**F**) Quantification of number of migrated cells. (**G**). Chick chorioallantoic membrane with CM-soaked filter disks. (**H**) Ranking of CAM assay (lowest =1 highest =15: average of 3 independent observers; box = interquartile range, whiskers +1%-99%) *N* = 6 per condition, performed with 2 batches of CM (each batch is represented by different symbols). EdU, 5-ethynyl-2′-deoxyuridine; DAPI, 4′,6-diamidino-2-phenylindole; CM, conditioned medium; CAM, chick chorioallantoic membrane; HUVEC, human umbilical vein endothelial cell.

**Figure 3.**
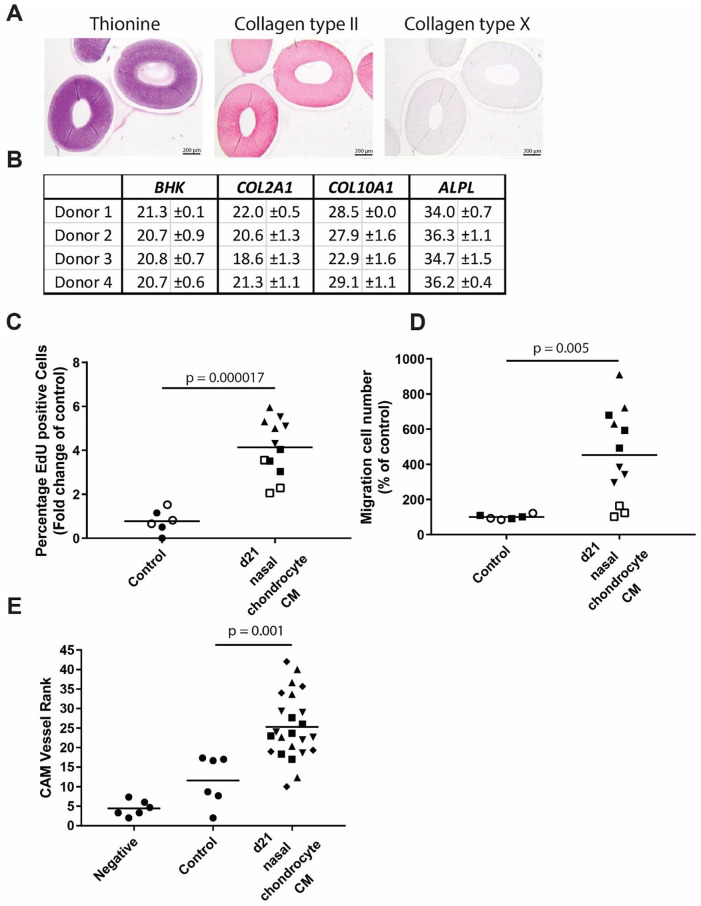
Chondrogenic redifferentiation of nasal septal chondrocytes releasate acts pro-angiogenic. (**A**) Histological and immunohistological staining of day 21 chondrogenically differentiated nasal chondrocytes for collagen type II, thionine, and collagen type X. (**B**) Quantitative polymerase chain reaction (qPCR) gene expression analysis of chondrocyte and hypertrophy marker genes showing the average and SD of 3 donors. Cutoff value for expression Ct = 35. (**C**) Quantification of number of proliferating cells. 2 experiments with a total of 4 separate donors done in triplicate. (**D**) Quantification of number of migrated cells. Two experiments with a total of 4 separate donors done in triplicate. (**E**) Vessel rank of chick chorioallantoic membrane (CAM) assay: higher rank = more pro-angiogenic. Four donors with *n* = 5.

The tissue-engineered cartilage from human articular chondrocytes stained positive for glycosaminoglycans and type II collagen, confirming cartilage tissue formation (**Fig. 2A**). Chondrogenic phenotype was further confirmed by high gene expression of chondrogenic marker *COL2A1* and a very low or absent expression of the hypertrophic markers *COL10A1* and *ALPL*. (**Fig. 2B**). To assess the effects of CM on specific aspects of angiogenesis, we performed an endothelial cell proliferation assay using EdU incorporation. The CM induced a 3.1-fold (±2.1) increase (*P* = 0.028) in the number of cells proliferating compared with the non-CM (**Fig. 2C** and **D**). To evaluate the effect of CM on endothelial cell migration we used a modified Boyden chamber assay. Exposure to CM resulted in 4.5-fold (±2.4) (*P* = 0.0004) increased number of migrated cells, indicating factors released by chondrogenically redifferentiated human articular cartilage derived constructs increase endothelial cell migration (**Fig. 2E** and **F**). The CM derived from the cartilage constructs attracted significantly (*P* = 0.006) more vessels than UCM in the CAM assay (**Fig. 2G** and **H**). The collected CM from tissue-engineered construct of human nasal septal chondrocytes (**Fig. 3B**) demonstrated a pro-angiogenic effect, similarly to the constructs from articular chondrocytes. In the *in vitro* assays the nasal chondrocyte construct releasate induced a 7.0-fold (±3.2) (*P* = 0.000017) increase in endothelial proliferation (**Fig. 3C**) and 4.5-fold (±2.7) (*P* = 0.005) increase in endothelial migration (**Fig. 3D**). Looking at the complete process of vessel formation in the CAM assay we observed significantly more vessels clearly directed toward the filter disks with CM of nasal septal chondrocytes than with the UCM (**Fig. 3E**). To confirm that the CAM assay was suitable to show anti-angiogenic effects we added a control with a blocking antibody of VEGFa. In summary, these results demonstrate that the releasate of chondrocytes derived from hyaline cartilage (articular and septal) is pro-angiogenic.

### Releasate of cartilage explants stimulated pro-angiogenic effect

**Figure 4.**
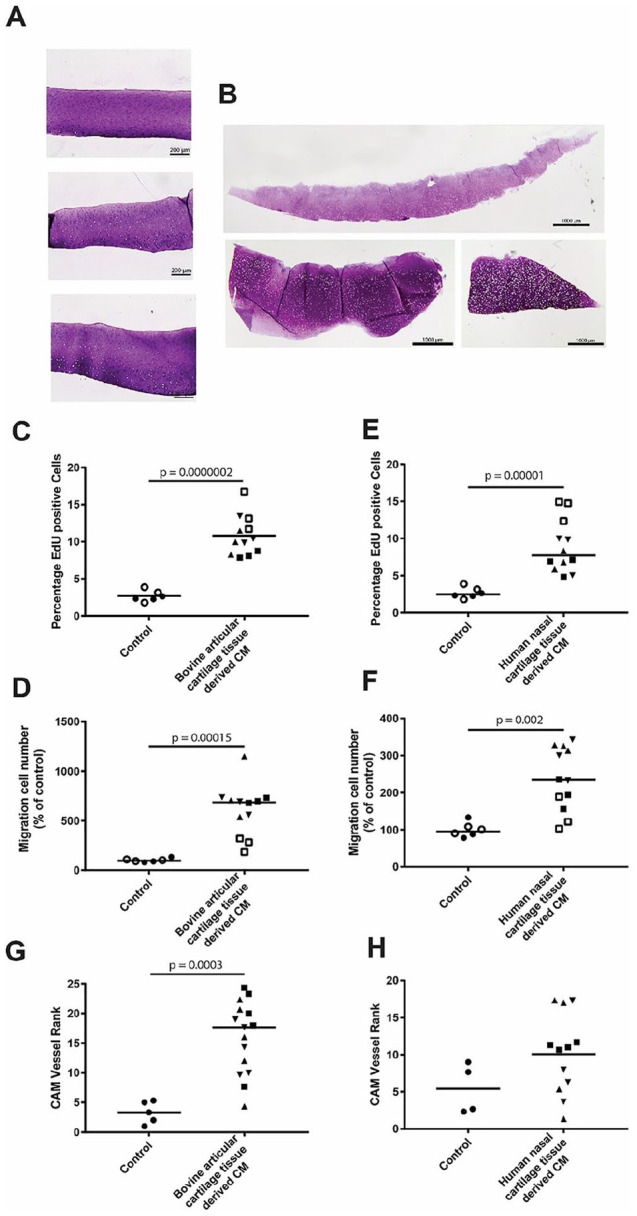
The releasates of healthy bovine articular cartilage and human nasal septal cartilage are pro-angiogenic. (**A**) Histology of bovine articular cartilage stained with thionine. (**B**) Histology of human nasal septal cartilage stained with thionine. (**C**) Endothelial cell proliferation with articular cartilage tissue–derived CM. (**D**) Endothelial cell migration with articular cartilage tissue derived CM. (**E**) Endothelial proliferation with nasal cartilage tissue–derived CM. (**F**) Endothelial cell migration with nasal cartilage tissue–derived CM. (**G**) CAM vessel rank with articular cartilage–derived CM. (**H**) CAM vessel rank with nasal cartilage tissue–derived CM. Higher rank = more pro-angiogenic. Statistical evaluation of the *in vitro* assays was done utilizing a linear mixed model with Bonferroni *post hoc* test (chondrocyte donors are represented with separate symbols, while the separate experiments are represented via the filling of the symbols). CM, conditioned medium; CAM, chick chorioallantoic membrane.

The CM of bovine articular cartilage and human nasal septal cartilage were evaluated *in vitro* for their effect on migration and proliferation of HUVEC and *in vivo* on a CAM assay (**Fig. 4**). The bovine articular cartilage–derived CM, increased proliferation 4.24-fold (±0.60) (*P* = 0.0000002) (**Fig. 4C**) and migration 6.1-fold (±2.36) (*P* = 0.0001) of endothelial cells (**Fig. 4D**). The nasal cartilage–derived medium led to a 3.43-fold (±0.95) (*P* = 0.00001) increase in endothelial proliferation (**Fig. 4E**) and a 6.06-fold (±2.36) (*P* = 0.002) increase in endothelial migration (**Fig. 4F**). Finally, the CAM assay confirmed this pro-angiogenic capacity of the releasate of cartilage explants as reflected by an increased attraction of vessels (**Fig. 4G** and **H**), albeit only with the bovine articular cartilage derived CM the effect was statistically significant.

**Figure 5.**
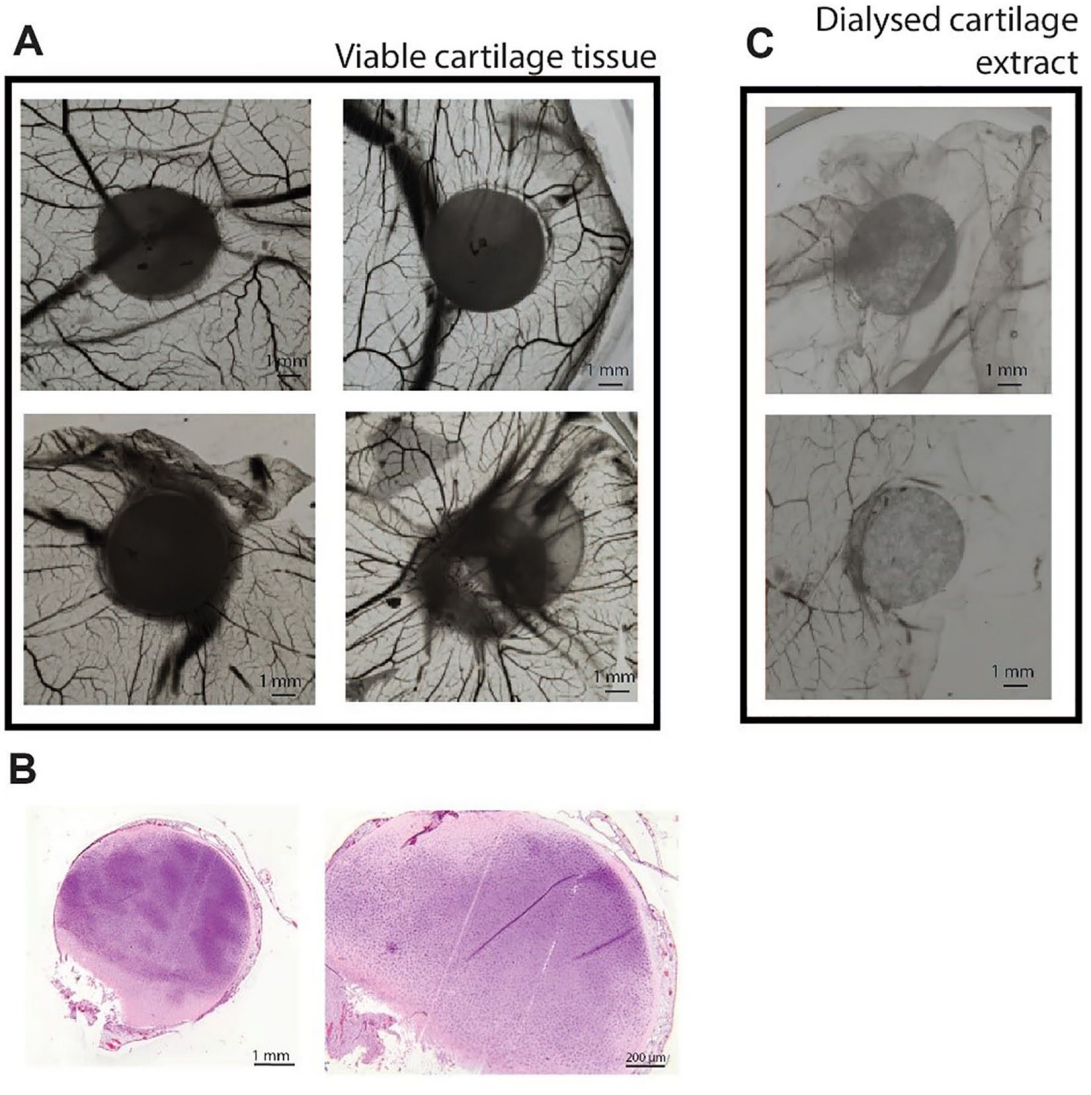
Bovine articular cartilage tissue shows a pro-angiogenic trend in the chick chorioallantoic membrane (CAM) assay. (**A**) Cartilage explants on the CAM, showing vessel attraction. (**B**) Representative sections of 1 of 8 cartilage explants after 7 days of incubation. (**C**) Dialyzed cartilage extracts on the CAM, showing inhibition of vessel formation. Pictures depict different donors of the shown conditions.

After the CM approach, we repeated the original set-up used by Eisenstein and colleagues^4^ using cartilage explants and cartilage tissue extracts. After a week of incubation of viable bovine articular cartilage explant on the CAM, we confirmed their observations that vessels did not penetrate in the cartilage (**Fig. 5A**); however, we saw a trend toward blood vessel attraction (**Fig. 5B**). Additionally, we extracted bovine articular cartilage with 4 M guanidine HCl. As in the experiments of Sorgente *et al*.,^27^ the dialyzed extract of bovine articular cartilage indeed inhibited blood vessel attraction in the CAM assay (**Fig. 5C**).

## Discussion

In this study, we demonstrated that the cartilage releasate is overall pro-angiogenic. This pro-angiogenic effect was proven with releasate from tissue-engineered cartilage from cultured human articular and nasal chondrocytes as well as cartilage explants of human nasal cartilage and bovine articular cartilage using *in vitro* assays for endothelial cell proliferation and migration as well as the CAM *in vivo* assay. Finally, we repeated the initial experiment described by Eisenstein *et al*. in which cartilage explants were used in the CAM assay and we confirmed their findings that cartilage tissue itself is not invaded by vessels. In fact, we could demonstrate that it did attract vessels. In addition, we confirmed that high salt extracts of cartilage were indeed anti-angiogenic. In combination with our gene expression data of adult cartilage we demonstrated a pro-angiogenic property of the cartilage releasate and showed that cartilage behaves like every other hypoxic tissue, however it has an additional anti-invasive property found in the tissue itself, possibly associated to cartilage matrix intrinsic properties or matrix bound factors.

We first determined the source of the common understanding that cartilage is anti-angiogenic. Early publications already described cartilage as a vessel-free tissue.^28^ The landmark series of publications contributing to the perception of cartilage as a tissue preventing vessel invasion was the 3-part series of “Tissues Resistant to Vascular Infiltration, I-III” published by the group of R. Eisenstein,^4,5,27^ in which cartilage was shown to prevent vessel invasion and cartilage extract was shown to prevent endothelial proliferation. This was further cemented by Folkman et al.^8,9^ demonstrating that cartilage, and its dialyzed high salt extract, has relevance for the development of anticancer drugs. In search for factors preventing angiogenesis to use in cancer therapy the line between anti-invasive factors and anti-angiogenic factor became blurred and led to cartilage being commonly accepted as being anti-angiogenic.^1,6-8,10-12,29-32^ We confirmed that cartilage explants are not invaded by blood vessels in the CAM assay and that a high salt extract of cartilage matrix inhibited blood vessel attraction. However, our results demonstrated that although not invaded, cartilage explants do attract vessels, indicating that its releasate is pro-angiogenic. The anti-angiogenic effects of high salt extract of cartilage are most likely due to released matrix-bound factors, which seem to be anti-angiogenic, as shown in the extract experiment (**Fig. 5E**), showing the anti-invasive characteristics of cartilage. Differing from the many publications that identified anti-angiogenic factors in cartilage,^12,33-36^ are the studies on osteoarthritis that showed pro-angiogenic properties of cartilage.^15,17,37-41^ Following these results, we found that pro-angiogenic properties of cartilage have been reported as early as the first publication of Eisenstein *et al*.^4^ Further studies supported this hypothesis, demonstrating the presence of VEGFa in mature articular cartilage, via gene expression or ELISA (enzyme-linked immunosorbent assay) analyses in the secretome.^17,18^ This is also reflected in the pro-angiogenic behavior observed in *in vitro* assays,^20,42^ although this behavior was ignored because the main goal of that study was to show an increased angiogenesis during OA. In our RNAseq dataset, we observed that, next to known anti-angiogenic genes, many pro-angiogenic genes were highly expressed.^22^ This comes with the caveat that there are currently more known pro-angiogenic than anti-angiogenic genes, which might skew the selection toward pro-angiogenic genes. The identified pro-angiogenic genes, however, are highly expressed and well-established mediators of vessel formation and attraction such as *VEGFa*, *HIF1a*, and *FGF1*, strengthening the conclusion of a potential pro-angiogenic effect of the cartilage releasate. Our selection of the top 1000 genes was made arbitrarily with the intention to focus on high expressed genes and only to get an insight into the distribution of angiogenesis regulating genes, explaining the absence of well-known anti-angiogenic genes which were lower expressed. Furthermore, the expression of many other genes leads us to hypothesise that the observed pro-angiogenic effect is explained by a combination of factors.

The most commonly used angiogenesis assays are based on the effects observed in HUVECs, which are commonly used as a model system to study subprocesses of vessel formation.^43^ Endothelial cell migration and proliferation mimic the first steps of the formation of a new vessel starting from an existing one, while the network forming assay or tube-forming assay displays the potential of completely new vessel formation. Altogether, these assays can provide a broad picture of the angiogenic effect of a specific factor or the whole releasate; however, they only reflect the effects of single cellular functions and do not reflect the cellular interplay needed in the complex process of vessel formation. To evaluate the effect of cartilage-released factors on a fully functional, mature vessel we utilized the CAM assay. This technique allows to observe the effect of different factors on the entire process of angiogenesis, from the disruption of a mature vessel to the formation, direction, and maturation of a new vessel. Overlapping multiple angiogenesis assays that span the entire vessel development process, we were able to confirm that the effects we observed *in vitro* are also observed *in vivo*. These results strengthened our conclusion that the cartilage releasate is pro-angiogenic.

We demonstrate that the releasate from both human articular and nasal chondrocytes-derived cartilage, was pro-angiogenic. Importantly, we previously shown that the cartilage derived from both types of chondrocytes remains stable (i.e., it does not get ossified) after *in vivo* implantation.^2,3^ The articular cartilage used for this study was obtained from total knee replacement surgeries, and although exclusively macroscopically, not diseased-affected sections were harvested, there are indications that this cartilage may contains cells with a hypertrophic phenotype.^44^ To address this issue, we also utilized nasal septal cartilage derived chondrocytes, a hyaline cartilage source, free of osteoarthritis, which confirmed the pro-angiogenic capacity of hyaline cartilage. To exclude a possible effect of TGFβ in the pro-angiogenic behaviour of the tissue engineered constructs,^21^ we also tested CM derived from the native cartilage tissue and demonstrated that both releasates induce a pro-angiogenic response. This effect was independent of the source of the tissue, as we tested both human nasal cartilage as well as bovine articular cartilage and when compared to previously published data it has a similarly pro-angiogenic effect as d21 chondrogenically differentiated.^26^ Experiments with cartilage explants on CAM further confirmed the secretion of factors that attracted blood vessels, clearly showing that the releasate of cartilage is not anti-angiogenic.

Besides showing that the releasate from cartilage and chondrocyte- derived constructs is pro-angiogenic, we also demonstrated that the apparent anti-angiogenic nature of cartilage is probably related to the matrix. With the common conception that articular cartilage turns pro-angiogenic during OA, a common approach has been to utilize anti-angiogenic treatment options.^15^ Our results suggest that the permissive invasion of blood vessels in OA might not be due to the presence of soluble factor(s), but instead might associated with specific cartilage matrix or matrix-bound factors. This could direct more attention to study the effects of cartilage integrity on blood-vessel invasion. For cartilage regeneration strategies it will be key to consider the replacement of this barrier function and to study the effects of the extracellular matrix for the possible role of extractable anti-angiogenic factors in cartilage repair and regeneration. Reproducing both on top of a mature chondrocyte phenotype, seem to be key to manufacture stable cartilage. This work is meant to supplement the already broad list of angiogenesis-related cartilage literature and to clarify the misunderstandings about the anti-angiogenic nature of cartilage that exist in the field. This information might be of value for further research on the embryonic development of cartilage and bone, the progression of OA and tissue engineering.

## Supplemental Material

sj-pdf-1-car-10.1177_19476035211047628 – Supplemental material for The Releasate of Avascular Cartilage Demonstrates Inherent Pro-Angiogenic Properties In Vitro and In Vivo Click here for additional data file. Supplemental material, sj-pdf-1-car-10.1177_19476035211047628 for The Releasate of Avascular Cartilage Demonstrates Inherent Pro-Angiogenic Properties In Vitro and In Vivo by Yannick Nossin, Eric Farrell, Wendy J.L.M. Koevoet, Frank Datema, Rodrigo A. Somoza, Arnold I. Caplan and Gerjo J.V.M. van Osch in CARTILAGE
