# Supplementary material for: The Releasate of Avascular Cartilage Demonstrates Inherent Pro-Angiogenic Properties In Vitro and In Vivo
Source: Cartilage. 2021 Sep 30;13(2 Suppl):559S–570S. doi: 10.1177/19476035211047628 (PMC8721614; doi:10.1177/19476035211047628)
Supplement: sj-pdf-1-car-10.1177_19476035211047628–Supplemental material for The Releasate of Avascular Cartilage Demonstrates Inherent Pro-Angiogenic Properties In Vitro and In Vivo [file sj-pdf-1-car-10.1177_19476035211047628.pdf]

Supplementary Materials

Sample processing, RNA isolation and RNA sequencing

In short, human adult AC samples, (2 males and 1 female aged 20-35-year, cause of death, gun shoot or drug overdose) were acquired (Articular Engineering, Northbrook, IL) and stored at -80C in RNAlater before processing. Additionally, human Mesenchymal Stem Cells (hMSCs) were isolated from bone marrow collected from the posterior-superior iliac crest of healthy adult donors ages 30–39 (n = 3) as previously described (Lennon, D. P., & Caplan, A. I. (2006). Isolation of human marrow-derived mesenchymal stem cells. Experimental Hematology, 34(11), 1604–1605. https://doi.org/10.1016/j.exphem.2006.07.014). For 21 days the hMSCs were cultured in cell aggregates in complete chondrogenic medium (DMEM HG supplemented with 1% ITS+, 0.1-µM dexamethasone, 1-mM sodium pyruvate, 120-mM ascorbic acid-2 phosphate, 100-mM nonessential amino acids, and 10-ng/ml TGF-β1).

Tissues were snap frozen in liquid nitrogen and pulverized by mechanical compression before static rotor homogenization in TRIzol (Life Technologies) at 4°C for 5 minutes. TRIzol-chloroform phase separation of nucleic acids preceded column isolation of RNA by Qiagen RNeasy Mini Kit. RNA quality was assessed by high sensitivity Agilent Bioanalyzer (Bioanalyzer RNA 6000 pico) and RIN scores ranged from 5.2 to 7.4. Low input total RNA (5ng-1000ng) was used for 150bp paired-end stranded RNA-seq at Novogene. Strand specific cDNA (insert size: 250-300bp) libraries were constructed using the NEBNext Ultra Kit for following poly-T oligo-attached magnetic bead purification. Quality assessed by insert size (Agilent 2100). Fragments per kilobases of transcript per 1 million mapped reads (FPKM) was used as a method of estimating gene expression levels, which takes into consideration the effects of both sequencing depth and gene length on counting of fragments. FPKM is calculated as follows: [number of fragments]/[(transcript length/1,000)/(total reads)/10^6)].

Of the three donors the FPMK values were averaged and sorted for the top 1000 expressed genes. These were further compared to three Gene Ontology and Uniprot databases regarding: angiogenesis (GO:0001525) 604 genes, negative regulation of angiogenesis (GO:0016525) 91 genes, as well as positive regulation of angiogenesis (GO:0045766) 218 genes. The identified genes are shown with each donor separated in Figure 1, with an average d21 chondrogenic differentiated MSCs FPMK (of 3 donors) as pro-angiogenesis control.

PCR Primer and probe sequences

Gene	Forward	Reverse	Probe
Alkaline Phosphatase, Biomineralization Associated (*ALPL)*	GGCAATAGCAGGTTCACGTACA	CGATAACAGTCTTGCCCCACTT	CCGGTATGTTTCGTGCAGCCATCCT
Collagen Type II Alpha 1 Chain (*COL2A1*)	GGCAATAGCAGGTTCACGTACA	CGATAACAGTCTTGCCCCACTT	CCGGTATGTTTCGTGCAGCCATCCT
Collagen Type X Alpha 1 Chain (*COL10A1*)	CAAGGCACCATCTCCAGGAA	AAAGGGTATTTGTGGCAGCATATT	TCCAGCACGCAGAATCCATCTGA
Glyceraldehyde-3-Phosphate Dehydrogenase (*GAPDH*)	GTCAACGGATTTGGTCGTATTGGG	TGCCATGGGTGGAATCATATTGG	TGGCGCCCCAACCAGCC
Hypoxanthine Phosphoribosyltransferase 1 (*HPRT1*)	TATGGACAGGACTGAACGTCTTG	CACACAGAGGGCTACAATGTG	AGATGTGATGAAGGAGATGGGAGGCCA
Ribosomal Protein S27 (*RPS27*)	TGGCTGTCCTGAAATATTATAAGGT	CCCCAGCACCACATTCATCA	(Syber-Green)
